# Application of the 3′ mRNA-Seq using unique molecular identifiers in highly degraded RNA derived from formalin-fixed, paraffin-embedded tissue

**DOI:** 10.1186/s12864-021-08068-1

**Published:** 2021-10-24

**Authors:** Jin Sung Jang, Eileen Holicky, Julie Lau, Samantha McDonough, Mark Mutawe, Matthew J. Koster, Kenneth J. Warrington, Julie M. Cuninngham

**Affiliations:** 1 Genome Analysis Core, Medical Genome Facility, Center for Individualized Medicine, Mayo Clinic, Stabile Research Building, 200 First Street SW, Rochester, MN 55905 USA; 2 Department of Laboratory Medicine and Pathology, Mayo Clinic, Rochester, MN USA; 3 Department of Internal Medicine, Division of Rheumatology, Mayo Clinic, Rochester, MN USA

**Keywords:** 3′ mRNA-Seq, UMI, FFPE, PCR amplification bias, Gene expression

## Abstract

**Background:**

Archival formalin-fixed, paraffin-embedded (FFPE) tissue samples with clinical and histological data are a singularly valuable resource for developing new molecular biomarkers. However, transcriptome analysis remains challenging with standard mRNA-seq methods as FFPE derived-RNA samples are often highly modified and fragmented. The recently developed 3′ mRNA-seq method sequences the 3′ region of mRNA using unique molecular identifiers (UMI), thus generating gene expression data with minimal PCR bias. In this study, we evaluated the performance of 3′ mRNA-Seq using Lexogen QuantSeq 3′ mRNA-Seq Library Prep Kit FWD with UMI, comparing with TruSeq Stranded mRNA-Seq and RNA Exome Capture kit. The fresh-frozen (FF) and FFPE tissues yielded nucleotide sizes range from 13 to > 70% of DV200 values; input amounts ranged from 1 ng to 100 ng for validation.

**Results:**

The total mapped reads of QuantSeq 3′ mRNA-Seq to the reference genome ranged from 99 to 74% across all samples. After PCR bias correction, 3 to 56% of total sequenced reads were retained. QuantSeq 3′ mRNA-Seq data showed highly reproducible data across replicates in Universal Human Reference RNA (UHR, R > 0.94) at input amounts from 1 ng to 100 ng, and FF and FFPE paired samples (R = 0.92) at 10 ng. Severely degraded FFPE RNA with ≤30% of DV200 value showed good concordance (R > 0.87) with 100 ng input. A moderate correlation was observed when directly comparing QuantSeq 3′ mRNA-Seq data with TruSeq Stranded mRNA-Seq (R = 0.78) and RNA Exome Capture data (R > 0.67).

**Conclusion:**

In this study, QuantSeq 3′ mRNA-Seq with PCR bias correction using UMI is shown to be a suitable method for gene quantification in both FF and FFPE RNAs. 3′ mRNA-Seq with UMI may be applied to severely degraded RNA from FFPE tissues generating high-quality sequencing data.

**Supplementary Information:**

The online version contains supplementary material available at 10.1186/s12864-021-08068-1.

## Background

Transcriptome profiling analysis is widely used in cancer research and clinical settings, such as drug discovery, diagnosis testing, and molecular biomarker discovery [1–3]. Formalin-fixed, paraffin-embedded (FFPE) tissue samples are the most commonly available clinical specimens resource having histopathology data for developing new molecular biomarkers in clinical research [4, 5].

High-quality RNA from fresh biological tissues is optimal to generate reliable transcriptome data. As FFPE samples are highly modified and fragmented with wide ranges of nucleotides, standard mRNA-Seq (poly-A selection) methods for transcriptome analysis are challenging [6, 7]; total RNA-Seq (with rRNA depletion) or RNA exome capture are the preferred methods [8–10]. However, total RNA-Seq using FFPE RNA is not generally consistent likely due to variation in RNA quality, with an abundance of intronic, intergenic, and rRNA reads and fewer exonic reads [7, 11]. Subsequently, fewer libraries are multiplexed for sequencing in each lane to yield sufficient reads than in standard mRNA-seq, leading to higher sequencing costs [7, 12]. While the RNA exome capture generates more exonic reads than total RNA-seq, the capture procedure incurs increasing library preparation costs. Recently developed 3′ mRNA-seq methods such as Tag-Seq [13], QuantSeq [14–16], and MACE RNA-Seq [17, 18] are now available. All three methods have similar procedures; however, QuantSeq has the most streamlined protocol, and all the reagents for library preparation are included in the kit. MACE RNA-Seq requires poly-A isolation before first stranded cDNA synthesis, while Tag-Seq is not available as a kit. This approach does not require RNA fragmentation before reverse transcription and only detects the 3′ end of the mRNA; thus, it may be used for degraded RNA samples, such as FFPE derived RNA, with a faster turnaround time and lower costs for library preparation and sequencing [19, 20]. 3′ mRNA-seq has been shown to yield data comparable with standard mRNA-seq in high-quality RNA and to be a reliable method for gene expression profiling in FFPE [15, 16, 18, 20]; however, performance in severely degraded FFPE samples has not yet been reported.

This study evaluates 3′ mRNA-Seq using the Lexogen QuantSeq 3′ mRNA-Seq Library Prep FWD Kit with unique molecular identifiers (UMI). The data are compared with TruSeq Stranded mRNA-Seq and RNA Exome Capture kit using Universal Human Reference RNA (UHR). RNA derived from fresh frozen (FF) and FFPE tissues with varying input amounts and nucleotide sizes range were used and compared with Exome Capture. Our results show that severely degraded FFPE RNA may be sequenced yielding accurate transcriptome profiling by 3′ mRNA-seq using UMI.

## Results

**Fig. 1:**
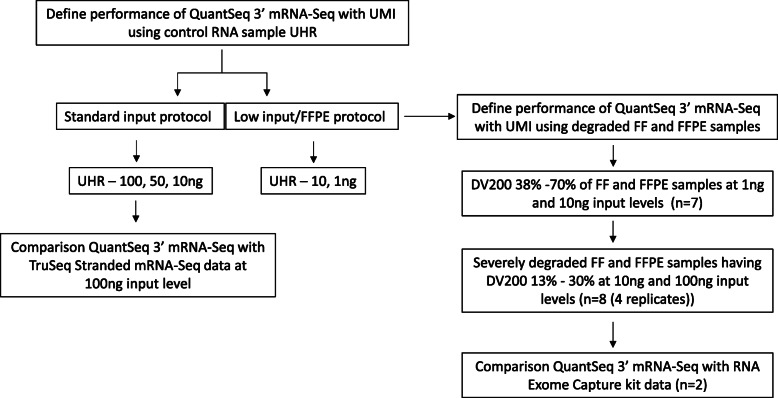
The overall experimental design

Figure 1 shows the design of this study. First, we evaluated the performance of Quantseq 3′ mRNA-Seq with UMI using a control RNA, UHR and compared with Tru-Seq stranded mRNA-seq. Next, we used FF and FFPE RNA samples, and severely degraded FFPE. For the latter, we included four replicates to evaluate reproducibility. These data were compared to Exome Capture, which is optimized for FFPE derived RNA. Samples used in this study had DV200 values ranging from 13 to > 70%, with input RNA between 1 ng and 100 ng and data for all samples in the study are included in Supplemental Data S1.

### QuantSeq 3′ mRNA-Seq performance using UHR and standard input and low input/FFPE protocols

**Fig. 2:**
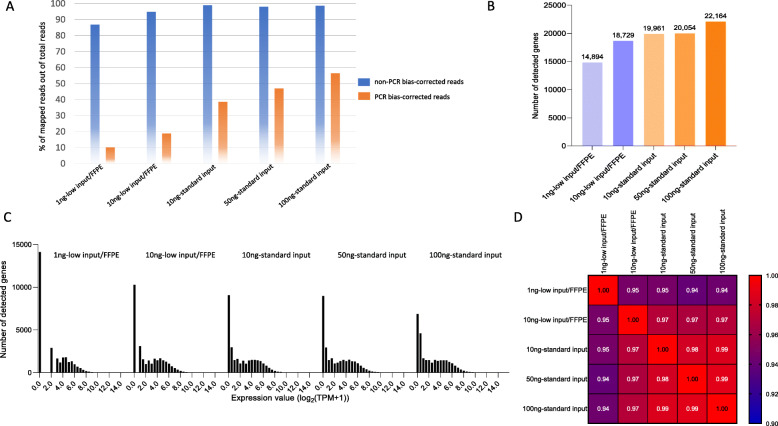
The PCR bias-corrected QuantSeq 3′ mRNA-Seq data in the UHR. A; Percentage of mapped reads out of total reads between standard input and low input/FFPE protocols by different input amounts. B & C; Total number of detected genes in the different inputs between protocols. D; Similarity matrix between the input amounts and protocols. Data were normalized by log_2_ (TPM + 1)

The QuantSeq 3′ mRNA-Seq kit has two protocols, standard input for high-quality RNA (> 10 ng) and low input/FFPE for degraded or small amounts of RNA (≤10 ng). We evaluated reproducibility with these two protocols using UHR. Total mapped reads were similar among the different input amounts and protocols (87–99% from total reads). However, the unique reads after PCR bias correction gradually dropped as total input RNA decreased (Fig. 2A, 56–10%). The total number of detected genes was ~ 15,000 to 22,000 genes (Fig. 2B), with the lower input/FFPE protocol showing fewer detected genes in the lower expressed genes (Fig. 2C). Overall, observed sample correlations were well matched within both protocols (standard input; R > 0.98, low input/FFPE; R > 0.94) and between protocols (R = 0.97, Fig. 2D).

### Comparison between QuantSeq 3′ mRNA-Seq and TruSeq stranded mRNA-Seq on UHR

**Fig. 3:**
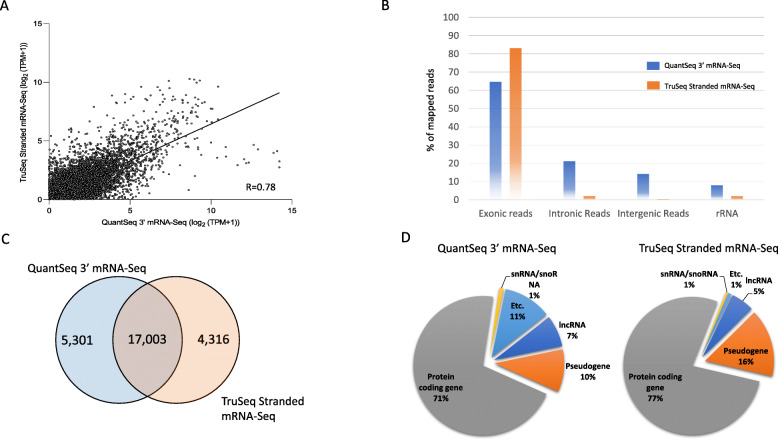
Data comparison between the QuantSeq 3′ mRNA-Seq and TruSeq Stranded mRNA-Seq kit. A; Correlation plot. Data were normalized by log_2_ (TPM + 1). Each dot constitutes a gene. B; Distribution of mapped reads. The incompatible paired-end reads (15%) were not reflected in the TruSeq Stranded mRNA-Seq data. C; Number of detected genes between two platforms. D; Percentage of mapped reads distribution by RNA biotypes

QuantSeq 3′ mRNA-Seq data using the standard input protocol was compared with Illumina TruSeq Stranded mRNA-Seq kit at 100 ng input level, which is the minimum RNA input amount recommended by Illumina. The correlation between the two protocols was moderate (R = 0.78, Fig. 3A), with the standard mRNA-Seq mapping more exonic region (65% vs. 83%) but fewer intronic region (21% vs. 2%), intergenic region (14% vs. 0.2%), and rRNA (8% vs. 2%, Fig. 3B). Both methods detected a similar number of expressed genes (22,304 and 21,319, Fig. 3C), and 17,003 genes were shared (Fig. 3C). QuantSeq 3′ mRNA-Seq data captured 71% of protein-coding genes from total detected genes and 77% in TruSeq Stranded mRNA-Seq (Fig. 3D).

### Performance of QuantSeq 3′ mRNA-Seq in moderately degraded RNA

**Fig. 4:**
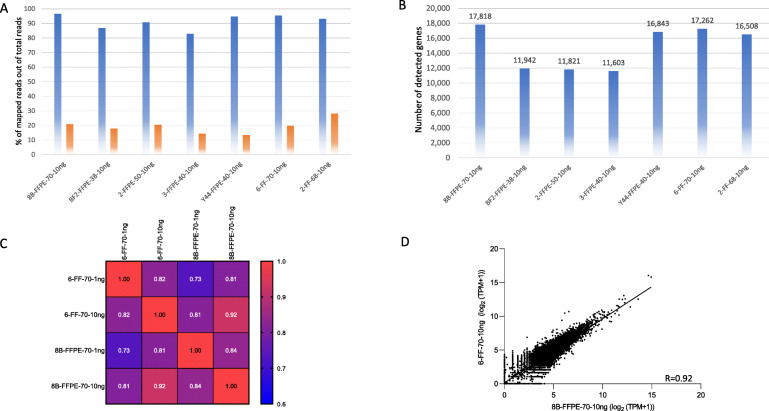
The PCR bias-corrected QuantSeq 3′ mRNA-Seq data in the degraded RNA (DV200 > 30%). A; Percentage of mapped reads out of total reads. Blue, non-PCR bias-corrected reads; Orange, PCR bias-corrected reads. B; Total number of detected genes. C; Similarity matrix in the paired FF and FFPE samples at 1 ng and 10 ng input. D; Correlation plot at 10 ng input between FF and FFPE samples. Samples 6-FF-70 and 8B-FFPE-70 are paired samples. Data were normalized by log_2_ (TPM + 1). 6-FF-70, 70% of DV200; 8B-FFPE-70, 70% of DV200;; 2-FFPE-50, 50% of DV200;; 3-FFPE-40, 40% of DV200;; 2-FF-68, 68% of DV200. Each dot constitutes a gene

Next, we evaluated QuantSeq 3′ mRNA-Seq using degraded RNA derived from FFPE and FF samples having > 30% (38–70%) of DV200 at 10 ng input. Total mapped reads were 83 to 97% but dropped to 13 to 28% after PCR bias correction (Fig. 4A). The total number of detected genes was 11,603 to 17,818 (Fig. 4B). Among the samples, there was one paired set of FF (6) and FFPE (8B) samples, and the agreement was 0.73 and 0.92 at the 1 ng and 10 ng input levels, respectively (Fig. 4C & D).

### Application QuantSeq 3′ mRNA-Seq for the severely degraded RNA

**Fig. 5:**
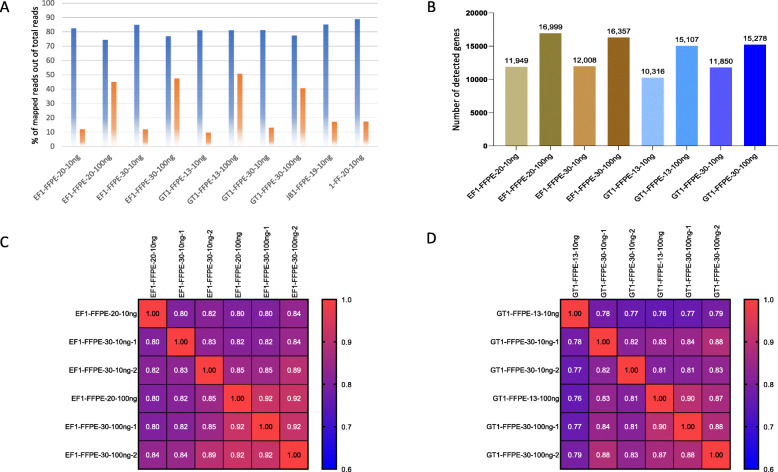
The QuantSeq 3′ mRNA-Seq data comparison using highly degraded RNA (DV200 ≤ 30%). A; Percentage of mapped reads out of total reads by different input amounts and average fragment size of RNA. Blue, non-PCR bias-corrected reads; Orange, PCR bias-corrected reads. B; Total number of detected genes in the different inputs and average fragment size of FFPE RNA. C & D; Similarity matrix at the 10 ng and 100 ng input amounts of EF1-FFPE-30 and GT1-FFPE-30. GT1-FFPE-13, 13% of DV200; GT1-FFPE-30, 30% of DV200; EF1-FFPE-20, 20% of DV200; EF1-FFPE-30, 30% of DV200; JB1-FFPE-19, 19% of DV200; 1-FF-20, 20% of DV200

To validate the performance of QuantSeq 3′ mRNA-Seq using highly degraded FFPE RNA with ≤30% (13–30%) of DV200 values, input amounts were increased to up to 100 ng to achieve sufficient unique reads after PCR bias correction. The unique reads at 10 ng input ranged from 10 to 17%, increased to ~ 40–50% after increasing the input amount to 100 ng (Fig. 5A). Along with increasing the unique reads, the total number of detected genes increased from 10,316 to 16,999 (Fig. 5B). Overall correlations in the 30% of DV200 FFPE samples were relatively high at the 100 ng input (EF1-FFPE-30, R = 0.92 & GT1-FFPE-30, R = 0.88), while moderate in the 10 ng input (EF1-FFPE-30, R = 0.83 & GT1-FFPE-30, R = 0.82, Fig. 5C & D). Similarly, 13 and 20% of DV 200 FFPE RNA showed good corerlation between samples at a 100 ng input level (EF1-FFPE, R = 0.92, GT1-FFPE, R = 0.87 & 0.90), and moderate correlation in the 10 ng input (EF1-FFPE, R = 0.80 & 0.84, GT1-FFPE, R = 0.77 & 0.79).

### Data comparison between QuantSeq 3′ mRNA-Seq and RNA exome capture kit in the severely degraded FFPE samples

**Fig. 6:**
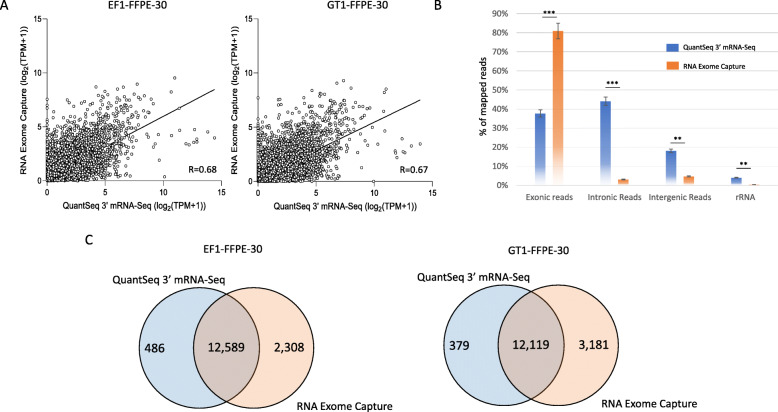
Data comparison between the QuantSeq 3′ mRNA-Seq and RNA Exome Capture. A; Correlation analysis. Data were normalized by log_2_ (TPM + 1). Each dot constitutes a gene. B; Distribution of mapped reads. Data are means of EF1-FFPE-30 and GT1-FFPE-30 samples from each kit ± SD.***, *p* < 0.001; **, *p* < 0.01. The incompatible paired-end reads (11%) were not reflected in the RNA Exome Capture data. C; Number of detected protein-coding genes between two platforms. EF1-FFPE-30, 30% of DV200; GT1-FFPE-30, 30% of DV200

The RNA exome capture method is designed for use with FFPE samples as standard mRNA-seq yields variable results; thus we compared RNA Exome Capture data with QuantSeq 3′ mRNA-Seqdata. Moderate correlation was observed with R = 0.68 (EF1-FFPE-30) and R = 0.67 (GT1-FFPE-30, Fig. 6A). The average exonic reads were 38% in the QuantSeq 3′ mRNA-Seq and 81% in the RNA Exome Capture kit, while intronic reads (44% vs. 3%), intergenic reads (19% vs. 5%) and rRNA reads (4% vs. 0.3%) were higher in QuantSeq 3′ mRNA-Seq than RNA Exome Capture kit (Fig. 6B). Total detected genes by RNA Exome Capture were 14,897 (EF1-FFPE-30) and 15,300 (GT1-FFPE-30), and shared 12,589 (EF1-FFPE-30) and 12,119 (GT1-FFPE-30), respectively. QuantSeq 3′ mRNA-Seq detected 13,075 (EF1-FFPE-30) and 12,498 (GT1-FFPE-30) genes (Fig. 6C).

## Discussion

Most mRNA-Seq studies use high-quality RNA from unfixed tissues or cells, and standard mRNA-Seq method is widely employed to investigate underlying biological differences. However, standard mRNA-Seq has a limitation when RNA is degraded with 3′ bias of the data and poor performance of library preparation. Several studies have suggested that a 3′ mRNA-Seq method may be a better option for such samples, as RNA degradation generally starts at the 5′ end [5, 16, 18]. In this study, we evaluated the performance of the QuantSeq 3′ mRNA-Seq using UMI for PCR bias correction to detect accurate gene expression data. Herein, we show 3′ mRNA-Seq using UMI to be an alternative option for the gene expression studies over a wide range of RNA derived from FFPE tissue.

To validate the performance of the QuantSeq 3′ mRNA-Seq with UMI, we first used UHR differing the input amount of RNA. Two protocols are available for QuantSeq 3′ mRNA-Seq, one standard input for higher quality RNA and one low input/FFPE protocol for FFPE derived or small amounts of RNA. Data were highly reproducible between the two methods. As expected, the unique mapped reads after PCR amplification error correction gradually decreased by RNA input amount. As each transcript molecule is barcoded with UMI before PCR amplification, the final data avoid PCR bias; thus, more accurate transcript counts are achievable even with 1 ng input amounts. However, TruSeq mRNA-Seq had better data quality with a higher proportion of exonic reads and less intron/intergenic and rRNA reads from total reads than QuantSeq 3′ mRNA-Seq. This difference may be related to the enrichment of alternative poly-A in the 3′ mRNA-Seq method [12, 21]. Also, it may be affected by the Internal priming of oligo dT primers on homopolymeric regions of transcripts, which generates erroneous reads during the first-strand cDNA generation [12]. Lastly, greater read depth in the TruSeq mRNA-Seq may increase exonic reads, while many 3′ RNA-seq reads correspond to poly-A sequences which when trimmed may also remove shorter reads and thus reduce relevant information [12]. In terms of data agreement, we observed a moderate correlation (R = 0.78), comparable to that reported by others using conventional mRNA-Seq and 3′ mRNA-Seq with UMI [22] or KAPA Stranded mRNA-Seq kit and the Lexogen QuantSeq 3′ mRNA-Seq kit without UMI [16]. This may reflect data differences related to longer transcripts count bias in standard mRNA-Seq and amplification error correction in the 3′ mRNA-Seq [18, 22]. The standard mRNA-Seq method requires a fragmentation step before reverse transcription with random hexamer to make cDNA, leading to more read counts per transcript, particularly from longer transcripts [16, 19, 23]. By contrast, the 3′ mRNA-Seq generates one read per transcript without fragmentation before reverse transcription, and PCR amplification error correction is reflected in the analysis [18].

The unique mapped reads and the total number of detected genes in the FFPE samples were dependent on RNA input, regardless of degradation levels. In this study, even severely degraded FFPE RNA may be used for QuantSeq 3′ mRNA-Seq with at least 100 ng input, and data were highly correlated with even in samples with ≤30% of DV200 values. Previously Turnbull et al. [20] reported more detected genes (25,610) using > 10-year-old FFPE samples, which used 500 ng input, suggesting that input amounts may be a more important factor than degradation level for increasing unique reads on QuantSeq 3′ mRNA-Seq. We observed a high correlation between paired FF and FFPE samples (R = 0.92) at the 10 ng input level. Recently, Boneva et al. [18] reported a high concordance rate between paired FF and FFPE samples (R^2^ = 0.88) using the MACE-Seq with UMI method at the 1000 ng level. This supports the tenet that 3′ mRNA-Seq method for FFPE samples is a reliable method for gene expression study.

RNA exome capture detects more fusion genes and alternatively spliced genes compared to standard mRNA-Seq and total RNA-Seq in FFPE samples [8, 9, 12]. Also, previous reports showed that gene expression quantification data is comparable with mRNA-Seq in high-quality RNA samples and total RNA-Seq in degraded samples [11, 24]. However, the direct correlation analysis between QuantSeq 3′ mRNA-Seq and RNA Exome Capture kit was not robust in this study. Like the TruSeq Stranded mRNA-Seq data above, data differences may relate to longer transcripts count bias and higher sequencing reads in the RNA Exome Capture and amplification error correction in QuantSeq 3′ mRNA-Seq. Although RNA Exome Capture data showed clear performance advantages over QuantSeq 3′ mRNA-Seq in the total number of genes captured, most of the protein-coding genes detected in the QuantSeq 3′ mRNA-Seq overlapped with RNA Exome Capture data. On the other hand, QuantSeq 3′ mRNA-Seq better quantifies gene expression. As Exome capture targets the coding region only, it generates more information to quantify gene expression [11, 12, 24]. However, compared to QuantSeq 3′ mRNA-Seq, RNA Exome Capture has a longer protocol, and the library preparation includes amplification before and after capture, which may affect data quality, particularly for more lowly expressed genes. Also, it captures only preselected RNAs and is only applicable for human samples [24]. While QuantSeq 3′ mRNA-Seq with UMI has a fast turnaround time, lower read depth but more accurate gene quantification, it reveals alternative poly-A sites, and allows more libraries to be multiplexed for sequencing [12, 16, 18]. Depending on project requirements, increasing read depth may be accomplished by altering multiplexing.

## Conclusions

This study evaluated QuantSeq 3′ mRNA-Seq using UMI in high-quality RNA comparing with TruSeq Stranded mRNA-Seq and with RNA Exome Capture using degraded RNA derived from FFPE tissue. We report that QuantSeq 3′ mRNA-Seq with PCR bias correction using UMI is a suitable method for gene quantification in both FF and FFPE RNAs. QuantSeq 3′ mRNA-Seq may be applied to even severely degraded RNA from FFPE tissues, generating high-quality sequencing data. QuantSeq 3′ mRNA-Seq using UMI is one means by which to investigate gene expression in a cost-effective manner, other approaches may yield more information and a greater number of detected genes, alternative splicing, and fusion genes. Thus, investigators should select the most suitable method based on the goals of the experiments and samples’ conditions because each platform has a different chemistry and sensitivity. Albeit, the QuantSeq 3′ mRNA-Seq using the UMI method provides an opportunity, particularly for gene expression analyses in severely degraded specimens, which may have not been feasible for RNA-Seq in the past.

## Methods

### RNA extraction from FF and FFPE samples

FFPE samples were cut to 10 μm thickness, and several tissue slices were put into a 1.5 ml tube. Xylene was added for deparaffinization, then total RNA was extracted with the Qiagen miRNeasy FFPE kit (Qiagen, CA, USA) following manufacturers’ protocol. Total RNA from fresh frozen (FF) Sample 6 was extracted using TRIzol (Thermo Fisher Scientific, MA, USA) following manufacturers’ protocol. UHR was purchased from ThermoFisher Scientific. Total RNA was quantified by Qubit and qualified by Agilent 2100 BioAnalyzer (Agilent Technologies, CA, USA). DV200 value (the percentage of RNA fragments > 200 nucleotides) was determined by 2100 expert software.

### Library generation

There are two protocols for the library preparation for the QuantSeq 3′ mRNA-Seq Library Prep Kit-FWD (Lexogen, Vienna, Austria). For the standard input protocol, UHR was incubated for 15 min at 42 °C to generate first-strand cDNA, and RNA was removed. The UMI second-strand synthesis mix was added to generate second-strand cDNA, followed by purification of double-stranded cDNA, and then PCR, using dual indices with 11 cycles for the library amplification was performed. UHR 10 ng and 1 ng, and all FFPE and FF samples were processed using the low input/FFPE protocol. Most processes are the same as standard input protocol for the low input/FFPE protocol, but incubation was increased to one hour for the first-strand cDNA and PCR was increased to 22 cycles for the library amplification.

For the standard mRNA-Seq library, the TruSeq Stranded mRNA-Seq library kit (Illumina, CA, USA) was used and followed manufactures’ protocol. Briefly, mRNA from 100 ng of UHR was isolated using mRNA isolation beads and fragmented for 4 min at 94 °C. The first-strand cDNA was synthesized at 42 °C, and the second-strand cDNA was synthesized at 16 °C for one hour with a second-strand marking buffer. Double strand cDNA was cleaned using DNA XP beads (Beckman Coulter, IN, USA), then A-tailed, ligated with index, amplified library with 15 cycles, and then the final library was cleaned using DNA XP beads.

For the RNA exome capture library, the TruSeq RNA Exome Capture kit (Illumina, CA, USA) was used and followed manufactures’ protocol. Briefly, 500 ng of highly degraded RNA was used for the first-strand cDNA synthesis at 42 °C. The second-strand cDNA was synthesized at 16 °C for one hour with a second-strand marking buffer. Double strand cDNA was cleanup with DNA XP beads, A-tailed, ligated with index, amplified library with 15 cycles, and then the final library was cleaned with DNA XP beads. cDNA library was quantified using Qubit and Agilent 2100 BioAnalyzer D1000 chip, and 200 ng of each library was pooled for exome enrichment and capture. After finishing the second enrichment, the pooled final libraries were amplified with 10 cycles and then the final library was cleaned using DNA XP beads.

The libraries were quantified by BioAnalizer 2100 system using the D1000 kit (Agilent, CA, USA) and Qubit dsDNA BR Assay kits (Thermo Fisher Scientific, MA, USA). All the libraries were sequenced 101 bp paired-end reads on Illumina HiSeq 4000 or MiSeq.

### Data analysis

For the 3′ mRNA-Seq data, ~ 1.5 to 8 million (M) of total reads were generated from each library. The Read 1 FASTQ files were uploaded into Partek Flow software (Partek Inc., MO, USA), and primary QC was performed. The UMI reads were identified, and adapter and poly A/T sequences were trimmed. The STAR (2.6.1d) [25] aligner was used to align reads to the human reference genome (hg38). After alignment, the final BAM files were quantified using the Partek E/M algorithm [26] after deduplicating UMIs by Ensembl annotations (Ensembl Transcripts release 92). For the standard mRNA-Seq and the RNA exome capture data, ~ 30 to 43 M pairs of total reads were generated from each library, and FASTQ files were uploaded into Partek Flow software. After primary QC was performed, the reads were aligned to the human reference genome (hg38) using STAR (2.6.1d) aligner. The final BAM files were quantified using the Partek E/M algorithm by Ensembl annotations (Ensembl Transcripts release 92). The aligned reads were normalized to TPM (Transcripts Per Kilobase Million) values and transformed log_2_ (TPM + 1) values. Pearson *R*-value was used for sample correlation analysis after PCR bias-corrected data. Protein-coding genes were used for the comparison between 3′ mRNA-Seq and RNA exome capture method. The two-tailed student’s t- test was used for statistical analyses.

## Supplementary Information

**Additional file 1: Supplemental Data S1.** Sample information and QC metrics of the QuantSeq 3′ mRNA-Seq with UMI.

## Data Availability

The sequencing datasets analyzed during the current study were deposited in the GEO repository (GSE 173506, https://www.ncbi.nlm.nih.gov/geo/query/acc.cgi?acc=GSE173506).

## Abbreviations

FFPE: formalin-fixed, paraffin-embedded
FF: Fresh Frozen
UHR: Universal Human Reference RNA
mRNA-Seq: mRNA sequencing
UMI: the unique molecular identifiers
TPM: Transcripts Per Kilobase Million

## References

[1] Yaeger R, Chatila WK, Lipsyc MD, Hechtman JF, Cercek A, Sanchez-Vega F, Jayakumaran G, Middha S, Zehir A, Donoghue MTA, You D, Viale A, Kemeny N, Segal NH, Stadler ZK, Varghese AM, Kundra R, Gao J, Syed A, Hyman DM, Vakiani E, Rosen N, Taylor BS, Ladanyi M, Berger MF, Solit DB, Shia J, Saltz L, Schultz N (2018). Clinical sequencing defines the genomic landscape of metastatic colorectal Cancer. Cancer Cell.

[2] Cancer Genome Atlas Research N (2013). Comprehensive molecular characterization of clear cell renal cell carcinoma. Nature.

[3] van Rheenen W, Diekstra FP, Harschnitz O, Westeneng HJ, van Eijk KR, Saris CGJ, Groen EJN, van Es MA, Blauw HM, van Vught PWJ, Veldink JH, van den Berg LH (2018). Whole blood transcriptome analysis in amyotrophic lateral sclerosis: a biomarker study. PLoS One.

[4] Hester SD, Bhat V, Chorley BN, Carswell G, Jones W, Wehmas LC, Wood CE (2016). Editor's highlight: dose-response analysis of RNA-Seq profiles in archival formalin-fixed paraffin-embedded samples. Toxicol Sci.

[5] Esteve-Codina A, Arpi O, Martinez-Garcia M, Pineda E, Mallo M, Gut M, Carrato C, Rovira A, Lopez R, Tortosa A (2017). A comparison of RNA-Seq results from paired formalin-fixed paraffin-embedded and fresh-frozen glioblastoma tissue samples. PLoS One.

[6] Bossel Ben-Moshe N, Gilad S, Perry G, Benjamin S, Balint-Lahat N, Pavlovsky A, Halperin S, Markus B, Yosepovich A, Barshack I, Gal-Yam EN, Domany E, Kaufman B, Dadiani M (2018). mRNA-seq whole transcriptome profiling of fresh frozen versus archived fixed tissues. BMC Genomics.

[7] Zhao W, He X, Hoadley KA, Parker JS, Hayes DN, Perou CM (2014). Comparison of RNA-Seq by poly (a) capture, ribosomal RNA depletion, and DNA microarray for expression profiling. BMC Genomics.

[8] Perry KD, Al-Lbraheemi A, Rubin BP, Jen J, Ren H, Jang JS, Nair A, Davila J, Pambuccian S, Horvai A (2017). Composite hemangioendothelioma with neuroendocrine marker expression: an aggressive variant. Mod Pathol.

[9] Huang W, Goldfischer M, Babayeva S, Mao Y, Volyanskyy K, Dimitrova N, Fallon JT, Zhong M (2015). Identification of a novel PARP14-TFE3 gene fusion from 10-year-old FFPE tissue by RNA-seq. Genes Chromosomes Cancer.

[10] Landolt L, Marti HP, Beisland C, Flatberg A, Eikrem OS (2016). RNA extraction for RNA sequencing of archival renal tissues. Scand J Clin Lab Invest.

[11] Cieslik M, Chugh R, Wu YM, Wu M, Brennan C, Lonigro R, Su F, Wang R, Siddiqui J, Mehra R, Cao X, Lucas D, Chinnaiyan AM, Robinson D (2015). The use of exome capture RNA-seq for highly degraded RNA with application to clinical cancer sequencing. Genome Res.

[12] Stark R, Grzelak M, Hadfield J (2019). RNA sequencing: the teenage years. Nat Rev Genet.

[13] Meyer E, Aglyamova GV, Matz MV (2011). Profiling gene expression responses of coral larvae (Acropora millepora) to elevated temperature and settlement inducers using a novel RNA-Seq procedure. Mol Ecol.

[14] Jarvis S, Birsa N, Secrier M, Fratta P, Plagnol V (2020). A comparison of low read depth QuantSeq 3′ sequencing to Total RNA-Seq in FUS mutant mice. Front Genet.

[15] Corley SM, Troy NM, Bosco A, Wilkins MR: QuantSeq. 3′ Sequencing combined with Salmon provides a fast, reliable approach for high throughput RNA expression analysis. Sci Rep 2019, 9(1):18895. 10.1038/s41598-019-55434-x PMC6906367 31827207

[16] Ma F, Fuqua BK, Hasin Y, Yukhtman C, Vulpe CD, Lusis AJ, Pellegrini M (2019). A comparison between whole transcript and 3′ RNA sequencing methods using Kapa and Lexogen library preparation methods. BMC Genomics.

[17] Zhernakov AI, Shtark OY, Kulaeva OA, Fedorina JV, Afonin AM, Kitaeva AB, Tsyganov VE, Afonso-Grunz F, Hoffmeier K, Rotter B, Winter P, Tikhonovich IA, Zhukov VA (2019). Mapping-by-sequencing using NGS-based 3′-MACE-Seq reveals a new mutant allele of the essential nodulation gene Sym33 (IPD3) in pea (*Pisum sativum* L.). PeerJ.

[18] Boneva S, Schlecht A, Bohringer D, Mittelviefhaus H, Reinhard T, Agostini H, Auw-Haedrich C, Schlunck G, Wolf J, Lange C (2020). 3′ MACE RNA-sequencing allows for transcriptome profiling in human tissue samples after long-term storage. Lab Investig.

[19] Tandonnet S, Torres TT (2017). Traditional versus 3′ RNA-seq in a non-model species. Genom Data.

[20] Turnbull AK, Selli C, Martinez-Perez C, Fernando A, Renshaw L, Keys J, Figueroa JD, He X, Tanioka M, Munro AF, Murphy L, Fawkes A, Clark R, Coutts A, Perou CM, Carey LA, Dixon JM, Sims AH (2020). Unlocking the transcriptomic potential of formalin-fixed paraffin embedded clinical tissues: comparison of gene expression profiling approaches. BMC Bioinformatics.

[21] Tian B, Manley JL (2017). Alternative polyadenylation of mRNA precursors. Nat Rev Mol Cell Biol.

[22] Xiong Y, Soumillon M, Wu J, Hansen J, Hu B, van Hasselt JGC, Jayaraman G, Lim R, Bouhaddou M, Ornelas L, Bochicchio J, Lenaeus L, Stocksdale J, Shim J, Gomez E, Sareen D, Svendsen C, Thompson LM, Mahajan M, Iyengar R, Sobie EA, Azeloglu EU, Birtwistle MR (2017). A comparison of mRNA sequencing with random primed and 3′-directed libraries. Sci Rep.

[23] Mandelboum S, Manber Z, Elroy-Stein O, Elkon R (2019). Recurrent functional misinterpretation of RNA-seq data caused by sample-specific gene length bias. PLoS Biol.

[24] Schuierer S, Carbone W, Knehr J, Petitjean V, Fernandez A, Sultan M, Roma G (2017). A comprehensive assessment of RNA-seq protocols for degraded and low-quantity samples. BMC Genomics.

[25] Dobin A, Davis CA, Schlesinger F, Drenkow J, Zaleski C, Jha S, Batut P, Chaisson M, Gingeras TR (2013). STAR: ultrafast universal RNA-seq aligner. Bioinformatics.

[26] Xing Y, Yu T, Wu YN, Roy M, Kim J, Lee C (2006). An expectation-maximization algorithm for probabilistic reconstructions of full-length isoforms from splice graphs. Nucleic Acids Res.

